# Novel Serial Positive Enrichment Technology Enables Clinical Multiparameter Cell Sorting

**DOI:** 10.1371/journal.pone.0035798

**Published:** 2012-04-24

**Authors:** Christian Stemberger, Stefan Dreher, Claudia Tschulik, Christine Piossek, Jeannette Bet, Tori N. Yamamoto, Matthias Schiemann, Michael Neuenhahn, Klaus Martin, Martin Schlapschy, Arne Skerra, Thomas Schmidt, Matthias Edinger, Stanley R. Riddell, Lothar Germeroth, Dirk H. Busch

**Affiliations:** 1 Institute for Medical Microbiology, Immunology and Hygiene, Technische Universität München, Munich, Germany; 2 Focus Group “Clinical Cell Processing and Purification”, Institute for Advanced Study, Technische Universität München, Munich, Germany; 3 Munich Center for Integrated Protein Science (CIPS-M) and Lehrstuhl für Biologische Chemie, Technische Universität München, Freising-Weihenstephan, Germany; 4 Stage Cell Therapeutics, Göttingen, Germany; 5 Clinical Cooperation Group “Antigen-specific Immunotherapy”, Helmholtz Center Munich (Neuherberg) and TUM, Munich, Germany; 6 Institute of Anaesthesiology, German Heart Center Munich, State of Bavaria and Technische Universität München, Munich, Germany; 7 Department of Hematology and Oncology, University Hospital Regensburg, Regensburg, Germany; 8 Program in Immunology, Fred Hutchinson Cancer Research Center, Seattle, Washington, United States of America; 9 DZIF - National Centre for Infection Research, Munich, Germany; Centro de Pesquisa Rene Rachou/Fundação Oswaldo Cruz (Fiocruz-Minas), Brazil

## Abstract

A general obstacle for clinical cell preparations is limited purity, which causes variability in the quality and potency of cell products and might be responsible for negative side effects due to unwanted contaminants. Highly pure populations can be obtained best using positive selection techniques. However, in many cases target cell populations need to be segregated from other cells by combinations of multiple markers, which is still difficult to achieve – especially for clinical cell products. Therefore, we have generated low-affinity antibody-derived Fab-fragments, which stain like parental antibodies when multimerized via *Strep*-tag and *Strep*-Tactin, but can subsequently be removed entirely from the target cell population. Such reagents can be generated for virtually any antigen and can be used for sequential positive enrichment steps via paramagnetic beads. First protocols for multiparameter enrichment of two clinically relevant cell populations, CD4^high^/CD25^high^/CD45RA^high^ ‘regulatory T cells’ and CD8^high^/CD62L^high^/CD45RA^neg^ ‘central memory T cells’, have been established to determine quality and efficacy parameters of this novel technology, which should have broad applicability for clinical cell sorting as well as basic research.

## Introduction

Cell therapy has proven to be highly effective for the treatment of a number of human diseases. For example, primary immunodeficiencies can be cured by hematopoietic stem cell transplantation (HSCT), and some patients with leukemia can be brought to complete remission by allogeneic HSCT alone, or combined with donor lymphocyte infusion (DLI). In some clinical settings, the adoptive transfer of virus-specific T cells can very effectively reconstitute immunity in immunocompromised patients, and prevent or treat life-threatening complications caused by cytomegalovirus reactivation [1], [2], [3] or lymphoproliferative diseases mediated by Epstein-Barr-Virus [4], [5]. Similarly, tumor-specific T cells, either from autologous tumor-infiltrating lymphocytes or engineered *in vitro*, are promising candidates for improved therapies for cancer [6].

Despite these interesting clinical observations, a broader transfer of cell therapy to clinical applications has remained a challenge. This is in part due to the problem that cell populations known to mediate clinical effects should best be enriched to high purities, since ‘unwanted’ contaminating cells can cause harmful and sometimes life-threatening side effects such as graft-versus-host-diseases (GvHD) mediated by alloreactive T cells. Very low numbers of adoptively transferred T cells can contribute to beneficial clinical effects [7], and similar rules will also apply to cell populations that mediate negative side effects. Therefore, providing the highest possible purities of well-defined cell preparations applicable for therapy is a key to making these promising treatments more effective and predictable, as well as to lower the risk of potential side effects.

Current methods for surface marker-mediated clinical cell purification usually rely on single parameters (e.g. CD34, MHC-multimers). However, for most cell populations – either purified directly *ex vivo* or expanded after *in vitro* cell culture – the use of a combination of different surface markers is necessary to truly segregate a defined subpopulation. For example, naturally occurring regulatory T cells (nTregs) represent a promising cell population for preventing acute GvHD after allogeneic HSCT [8], [9], [10] or the development of several autoimmune diseases [11], [12]. Regulatory T cells lack a single cell-specific surface marker, therefore most current protocols for enrichment of nTregs from primary blood specimens or *in vitro*-expanded sources employ multiple markers such as CD4 and the constitutively expressed high-affinity IL-2 receptor α-chain (CD25), to reduce heterogeneity. However, CD25 is also expressed on many non-regulatory cells, which include recently activated effector and memory T cells. Therefore, complex combinatorial staining patterns, comprising combinations of CD4, CD25, CD127 and CD45RA [13], [14], have been necessary to more precisely identify and separate this clinically relevant T cell subset. Similarly, T cells with a central memory phenotype (T_CM_) can only be segregated from other subsets by combinatorial surface marker expression patterns (CD3^+^, CD62L^+^, CD45RO^+^, CD45RA^−^). T_CM_ exhibit superb characteristics for adoptive T cell transfer due to their longevity *in vivo*, and cell cultures derived from highly purified T_CM_ have enhanced persistence after adoptive transfer [15]. Thus, the T_CM_ subset is an interesting source for adoptive transfer of primary (unmanipulated) T cells as well as for genetic engineering to express defined recombinant T cell receptors (TCRs) or chimeric antigen-receptors (CARs) prior to administration [16], [17].

Currently available surface marker-based cell separation techniques usually utilize paramagnetic beads. Thereby, positive enrichment strategies using directly labeled target populations give the highest yields and purities. Yet, a more stringent purification that requires the combination of several markers cannot be achieved by positive selection alone. Therefore, protocols combining initial depletion of most unwanted cell populations by negative selection followed by a final positive enrichment step have been developed, which in some cases provide quite pure cell products. Unfortunately, the large number of surface markers and corresponding clinical-grade reagents required for depletion strongly interferes with the feasibility of this strategy for clinical applications. In addition, after positive selection, both the labeling reagents and the beads usually remain on the enriched cells, potentially manipulating the isolated cell population or negatively impacting its functionality and/or viability [18], [19]. Particularly with respect to clinical cell sorting, lingering cell labels pose substantial regulatory hurdles for the treatment of patients with such cell products. In order to circumvent the problems of positive selection, many clinical cell-processing procedures have been changed to exclusively employ depletion reagents. Unfortunately, under such conditions target cell purities are often poor, and depletion methods commonly require a complicated cocktail of different antibodies, making their production and application laborious and expensive.

We have recently introduced the MHC-*Strep*tamer technology for the positive selection of antigen-specific CD8^+^ T cells [18]. This simple cell purification procedure allows the release and complete removal of all components of the selection marker from the purified cell population. The major advantage of this strategy is that positive cell purification can be applied to obtain highly enriched antigen-specific T cells that have been classified by regulatory authorities as minimally manipulated cell products, which facilitates substantially the generation and usage of such cells for clinical applications. We hypothesized that the removal of positive selection labels after a purification step could be applied to any cell surface marker, allowing purification of cell populations that can only be defined by multiple parameters via serial positive enrichment. Here we describe a novel method, so-called Fab-*Strep*tamers, which fulfills exactly these criteria.

## Materials and Methods

### Blood samples

Fresh PBMCs were generated from either peripheral blood or buffy-coats by centrifugation over Biocoll separating solution. Peripheral blood was obtained from healthy adult donors of both sexes at the Institute of Medical Microbiology, Immunology and Hygiene (Technical University Munich), and buffy-coats were obtained from autologous male or female blood donors (17–82 years old) at the Institute for Anesthesiology, German Heart Centre Munich (State of Bavaria and Technical University Munich). Written informed consent was obtained from the donors, and usage of the blood samples was approved according to national law by the local Institutional Review Board (Ethikkommission der Medizinischen Fakultät der Technischen Universität München).

### Production of Fab-multimers (Cloning, Expression, Purification, Multimerization)

Monomeric Fab-fragments originating from monoclonal antibodies [20] were generated by gene synthesis (Invitrogen) or by PCR-based cloning of the variable region from hybridomas [21] (parental clones αCD3: OKT3; αCD4: 13B8.2; αCD8: OKT8; αCD25: FRT5; αCD62L: DREG56; αCD45RA: MEM56). After generation of cDNA, the hypervariable sequences of heavy and light chains were amplified as described before [21] and verified by sequencing. The obtained variable (V) domains from the heavy (V_H_) and light (V_L_) chains were cloned on a pENTRY-IBA50 StarGate vector allowing the combination with sequences coding for the constant domains of human subclass IgG1/κ [22] in a subsequent recombination step. The heavy chains were carboxy-terminally fused with a OneSTrEPtag affinity tag (IBA). All combinatorial cloning was done using the StarGate cloning system (IBA) with fusion vectors adapted for periplasmatic Fab expression. The cistron organization has been described before [23]; a schematic overview is depicted in Fig. S1. In some cases mutagenesis PCR was applied to introduce amino acid substitutions within the non-hypervariable framework regions. Following cloning, both the chimeric heavy and the light chain were periplasmatically expressed in *E. coli* K-12 strain JM83 allowing protein folding, disulfide bond formation and assembly of the Fab heterodimer [24]. Fab fragments were produced in 2L LB shaking cultures supplemented with 100 µg/ml ampicillin (Amp). Recombinant protein was harvested 3 hours post anhydrotretracyclin (500 µg/ml) induced gene expression, periplasmic extract was prepared as described before [25] and Fab-fragments were purified by *Strep*-tag/*Strep*-Tactin affinity chromatography via a *Strep*-Tactin Superflow column (IBA) and stored in PBS pH 7.5 [26].

One µg Fab-multimer consisting of monomeric Fab-OneSTrEPtagged fragment and *Strep*-Tactin labeled either with phycoerythrine or allophycocyanin was used to stain up to 5×10^6^ cells.

### FACS Analysis

For FACS analysis, 5×10^6^ PBMCs were incubated with the multimeric Fab-*Strep*-Tactin-complexes (Fab-multimers) for 20 minutes at 4°C. Combined antibody stainings were performed by concomitant application of the respective antibodies: anti-CD3 (OKT3), anti-CD4 (OKT4), anti-CD8 (OKT8), anti-CD45RA (HI100) and anti-CD62L (DREG56, all from eBiosciences), anti-CD25 (ACT-1), anti-CD45RO (UCHL1) and anti-CD69 (FN50, all from Dako Cytomation) and anti-CD30 (Ber-H83, Becton Dickinson). After surface staining, cells were washed and subsequently stained with propidium iodide (Molecular Probes) for live/dead cell discrimination.

For intranuclear FoxP3 staining, isolated cells were incubated with EMA, permeabilized and fixated as recommended by the manufacturer (eBioscience). Staining with anti-FoxP3 antibody (PCH101, eBioscience) was performed for 30 min at 4°C. Data were collected by flow cytometry on a CyAn ADP Lx (Beckman Coulter) and analyzed with FlowJo software (TreeStar).

### Sequential magnetic enrichment

For the sequential magnetic enrichment of CD4^+^CD25^+^CD45RA^+^ triple-positive cells, 1×10^8^ PBMCs were first incubated with *Strep*-Tactin-functionalized (in total 15 µg *Strep*-Tactin was used) magnetic beads (1 µm approximate diameter) coated overnight with the reversible CD4 Fab-monomers. Subsequently, CD4^+^ cells were isolated by retention on a NdFeB permanent magnet (Q-60-30-15-N40 from Supermagnete, Gottmadingen, Germany). Non-retained cells were removed, and the magnet was removed and then reapplied after washing the retained cells in 5 ml heparinized PBS containing 0.5% BSA (w/v). This procedure was repeated five times and the CD4^+^ cells were then completely liberated from the beads and Fab-fragments by addition of 1 mM D-biotin (applied twice) and washing (twice, including a 10 min incubation time). In two further consecutive enrichment steps, CD25- or CD45RA-coated beads were used to isolate either CD4^+^CD25^+^ or triple positive cells from the pre-enriched CD4^+^ or CD4^+^CD25^+^ cell pool respectively, and further processed as described above. CD8^+^ T_CM_ (CD8^+^CD62L^+^CD45RA^neg^) were enriched with CD8- and CD62L Fab-functionalized beads as described above. CD45RA Fab-beads were used as last step to deplete CD45RA-positive cells in the CD8^+^CD62L^+^ pre-enriched preparation. For determination of the final yields, absolute cell numbers were determined by counting appropriate dilutions of the final positive fraction in a Neubauer counting chamber. Live/dead discrimination was performed using Trypane blue staining.

### Suppression assay

Suppression of effector T cell proliferation was determined in a CFSE dilution assay after polyclonal anti-CD3 stimulation (0.5 µg/ml, clone OKT3) at a Treg∶T responder cell ratio of 2∶1. For the proliferation assay effector cells were labeled with 2.5 µM CFSE (Molecular Probes), and 2×10^4^ effector T cells (along with 4×10^4^ Treg cells and 1×10^5^ autologous irradiated feeder cells) were plated in 96-well plates in supplemented RPMI (Gibco). After 5 days, CFSE signal dilution was measured on a CyAn ADP Lx cytometer (Beckman Coulter).

## Results

### Principle of reversible Fab-multimer staining

**Figure 1:**
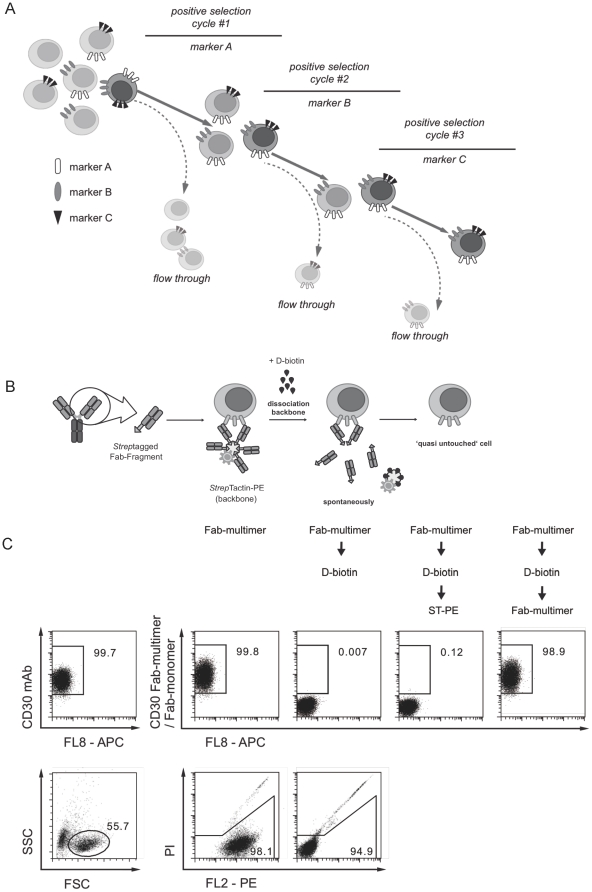
Principle of reversible Fab-multimer staining. (a) Schematic overview of sequential positive cell enrichment addressing a subset within complex cell mixtures that can only be defined by combined expression of three markers ‘A’, ‘B’, and ‘C.’ Three serial enrichment steps allow exclusive purification of the target population. (b) Illustration of the basic principle of reversible Fab-multimers. Low affinity-modified Fab fragments are reversibly multimerized by Streptag-*Strep*-Tactin complexation. Subsequent treatment of stained cells with D-biotin mediates destruction of the Fab-multimer complex and results in spontaneous dissociation and complete removal of all (monomeric) components from the target cell surface. (c) Experimental proof-of-concept for fully reversible Fab-multimer staining. CD30-positive cells from the L1236 cell line were stained with either a monoclonal antibody (left dot plot) or cognate PE-labeled Fab-multimers and analyzed either before (second left column) or after treatment with D-biotin (middle column). Remaining Fab-monomers were then detected after subsequent washing steps using fresh PE-labeled *Strep*-Tactin (second right column). Secondary Fab-multimer staining of reversibly stained cells served as control (right column). Only live (PI^negative^) L1236 cells are shown (dead cell gating is shown in the bottom row). Numbers in dot plots indicate the percentage of cells within gates.

We hypothesized that multiparameter cell sorting by serial positive enrichment could be achieved if the specific cell label used for each enrichment step could be removed before entering the next purification cycle (Fig. 1a). Since most approaches for specific labeling of cell surface markers rely on antibody staining, we developed reversible staining probes based on existing reagents. Antigen specificity of an antibody is determined within the variable region of its Fab portion, which contains identical binding regions displayed in 2 (IgG, IgA, IgE) or 10 (IgM) copies, indicating the role of multiple interactions with the antigen to enhance binding avidity. We speculated that in cases where monomeric Fab-molecules are of such low affinity that they alone cannot stably bind to a given antigen, the associated avidity gain by multimerization should enable such probes to be used for specific cell labeling [27]. Furthermore, if multimerization of such Fab-reagents could subsequently be reversed by targeted disruption of the complex, the low-affinity Fab-monomers should spontaneously dissociate from the cell surface, leaving purified cells fully liberated from all labeling components (Fig. 1b), thereby allowing the transfer of these cells to additional (serial) positive enrichment steps.

To demonstrate first ‘proof of concept’ of this reversible Fab-multimer strategy, we used the well-characterized huHRS3 Fab-fragment directed against CD30 [24]. This fragment was derived from the parental monoclonal antibody HRS3 by grafting the murine CDRs onto a human immunoglobulin consensus sequence, which initially resulted in a functional binding site for CD30 but was accompanied by a substantial loss in affinity (K_D_ = 278±61 nM from equilibrium analysis) [24]. In order to introduce a multimerization site, the heavy chain of the Fab-fragment was genetically fused to the OneSTrEP-tag sequence (*Strep*-tag), and expressed together with the light chain in the periplasm of *E. coli* [24]. The functionally assembled Fab-fragments were then purified by affinity chromatography on a *Strep*-Tactin resin and subsequently multimerized in the presence of phycoerythrin-labeled *Strep*-Tactin. Notably, this interaction is reversible upon addition of D-biotin (or its derivatives) as a competing ligand (Fig. 1b) [26].

As shown in Fig. 1c, CD30 Fab-multimers brightly stained cells from the CD30-expressing L1236 cell line in a manner identical to that of a commercially available anti-CD30 antibody. In order to analyze the subsequent removal of cell-bound Fab-multimers, cells were first treated with D-biotin to disrupt the binding between the *Strep*-tag on the Fab-fragment and the *Strep*-Tactin “backbone”. FACS analysis of D-biotin-treated cells showed no remaining PE-conjugated *Strep*-Tactin on the cells, indicating efficient and complete dissociation of multimeric complexes. To visualize potential residual cell-bound Fab-monomers, D-biotin-treated cells were washed and subsequently incubated with fresh (uncomplexed) *Strep*-Tactin-PE. Based on this sensitive detection method, we could not identify any remaining Fab-fragments on the cell surface, indicating that the cell labeling was fully reversible. Efficient re-staining of liberated cells with anti-CD30 Fab-multimers also excluded the possibility that remaining D-biotin interfered with *Strep*-Tactin-PE rebinding through blockade of *Strep*-tag binding sites in the subsequent detection steps. These data demonstrate that multimerization of low affinity Fab-monomers can be used to generate fully reversible staining probes.

### Engineering of reversible Fab-multimers by mutations

**Figure 2:**
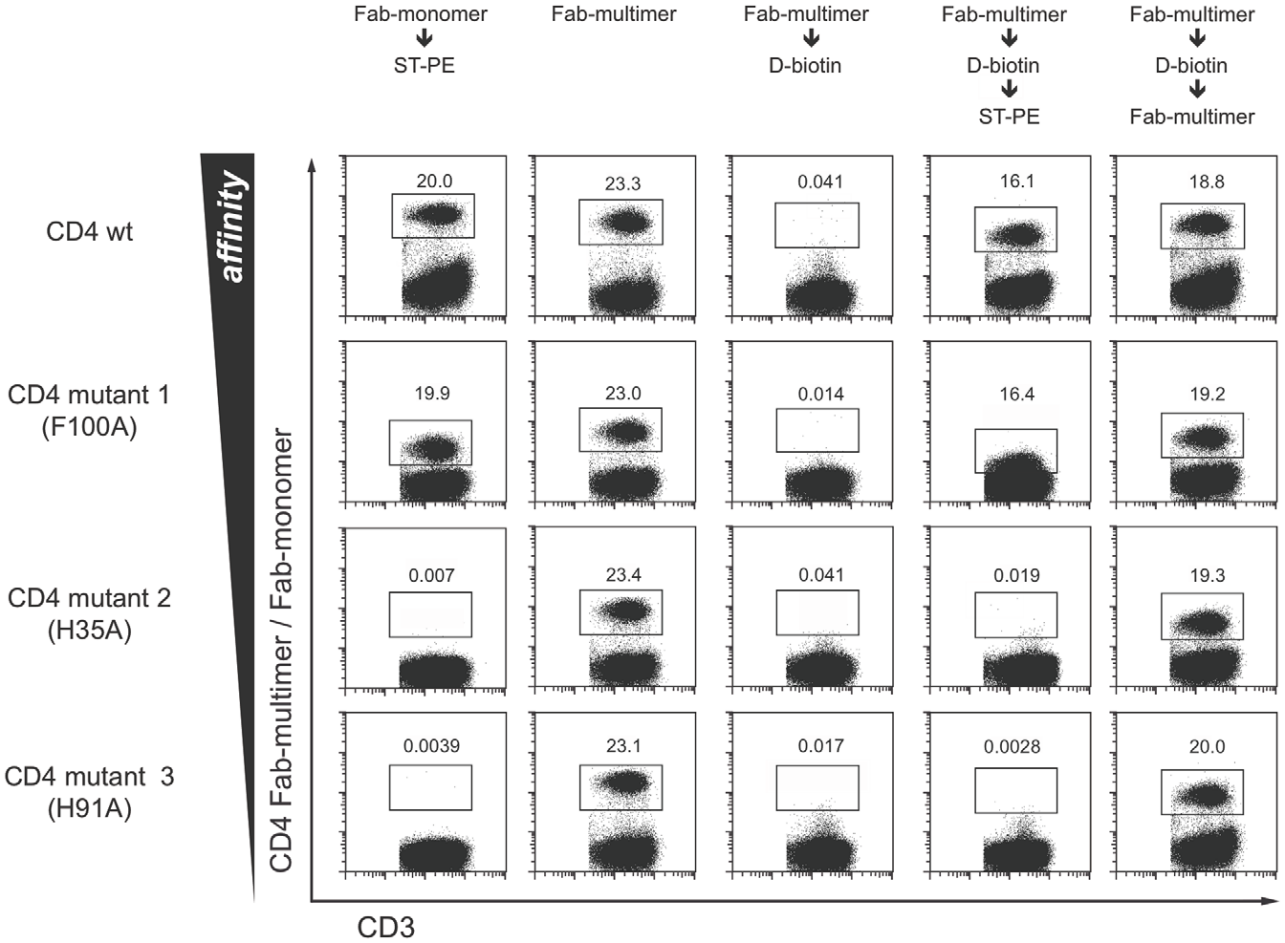
Binding characteristics required for reversible Fab-multimer staining. FACS analysis of anti-CD4 Fab-multimer staining with different anti-CD4 Fab mutants with decreasing affinities. PBMCs were stained with the respective PE-labeled anti-CD4 Fab-multimers and analyzed either before (second column) or after treatment with D-biotin (third column). Remaining Fab-monomers were then detected after subsequent washing steps using (uncomplexed) PE-labeled *Strep*-Tactin (fourth column). Secondary Fab-multimer staining of reversibly stained cells served as control (right column). Alternatively, cells were incubated with monomeric Fab-fragments, washed and subsequently analyzed after staining with *Strep*-Tactin (left-most column). Live CD3^+^ T cells are shown. Numbers in the dot plots indicate the percentage of cells within gates.

In order to generate reversible staining reagents from a broad spectrum of parental antibodies or for different antigens, it is necessary to lower binding when monomeric Fab-fragments are of too high affinity. In order to test whether this can be generally achieved by the introduction of specific amino acid exchanges, we generated recombinant Fab-fragments derived from an anti-CD4 antibody with a known high Fab binding affinity (K_D_ value of 29 nM [13B8.2], as previously determined by surface plasmon resonance [20]). In addition, we generated recombinant Fab-*Strep*-tag fusion proteins containing mutations that have been described to broadly change affinities (K_D_s ranging from low affinity, K_D_ = 16.9 µM [mutant 3], to intermediate K_D_s of 0.8 µM [for mutant 1] and 6.3 µM [mutant 2]) [20]. Site directed amino acid substitutions were only introduced within the framework of heavy and light chain variable regions without targeting the highly variable CDR regions. For staining and dissociation experiments, we incubated 5×10^6^ PBMCs with the respective anti-CD4 Fab-multimer complexes or with the corresponding Fab-monomers (the latter not complexed with *Strep*-Tactin). Whereas the Fab-mutants showed essentially equal staining signals when multimerized, only the 13B8.2 Fab-fragment and to a minor extent, mutant 1, which exhibited higher affinities, were able to bind to their antigen in a monomeric state (Fig. 2, first column). After D-biotin-mediated disruption of the multimeric complexes, the cells were probed for residual surface-bound Fab-fragments. As expected from the monomer staining experiments, no remaining Fab-monomers could be detected for mutants 2 and 3, whereas substantial residual cell surface presence of the wildtype and, to a minor extent mutant 1 was observed. For mutants 2 and 3, cells could be efficiently re-stained using secondary Fab-multimer labeling.

In addition to flow cytometry-based analysis, complete removal of Fab-multimers was assessed by western blot analysis. Confirming the highly sensitive FACS results (Fig. S2), we could not detect Fab-fragments in either the supernatant or the pellet fraction after cell lysis, ruling out any significant internalization of surface-bound reagents during the staining and release procedure.

In summary, these data demonstrate that engineering of Fabs with variable binding affinities can be achieved by the introduction of amino acid changes. Besides the shown CD4 Fab-fragments, fully reversible Fab-fragments for all other target antigens used in this manuscript could be generated as described above. In rare occasions (monomeric) wildtype Fab-fragments already displayed a sufficiently low intrinsic affinity resulting in their spontaneous release. Fab-fragments – even when modified to have quite low binding affinities – can preserve superb staining qualities as multimers in a manner similar to the parental antibodies. Most important and in contrast to monoclonal antibodies, low affine Fabs can be completely removed from the surface of labeled cells.

### Sequential magnetic enrichment of central memory T cells

Reversible Fab staining was developed to enable positive enrichment of cell populations that need to be defined via multiple surface markers. To test this, we decided to establish a protocol for the enrichment of CD8^+^ central memory T cell (T_CM_), which are characterized by co-expression of CD8, CD62L and CD45RO whilst being negative for CD45RA. An initial attempt by our laboratories to enrich T_CM_ used currently available non-reversible reagents, and involved depletion of cells carrying a number of exclusion markers (CD4, CD14, CD45RA) followed by a single positive enrichment step for CD62L expressing cells (Fig. S4). The yield of T_CM_ (% of the number of purified target cells in relation to the number of target cells in the original sample) in the final product averaged 25%, which is expected when considering a cell loss of approximately 50% for each processing step. Interestingly, the yields varied substantially from donor to donor, which might be explained by variable expression patterns (from bright to intermediate to low/negative) of some markers, especially CD62L. Unfortunately, the purities of T_CM_ in the final cell product were suboptimal (mean 36%), mainly due to contaminating CD13^+^ basophilic granulocytes of which the majority expresses CD62L but lack the three markers that were targeted with the depleting antibodies (Fig. S4). Therefore, further improvement of this protocol for clinical T_CM_ purification would require at least the addition of anti-CD13 antibody into the depletion cocktail. For clinical applications, all antibodies (here at least 5) have to be generated under GMP conditions, illustrating the financial, technical and regulatory limitations of current technologies even for this relatively straightforward application.

**Figure 3:**
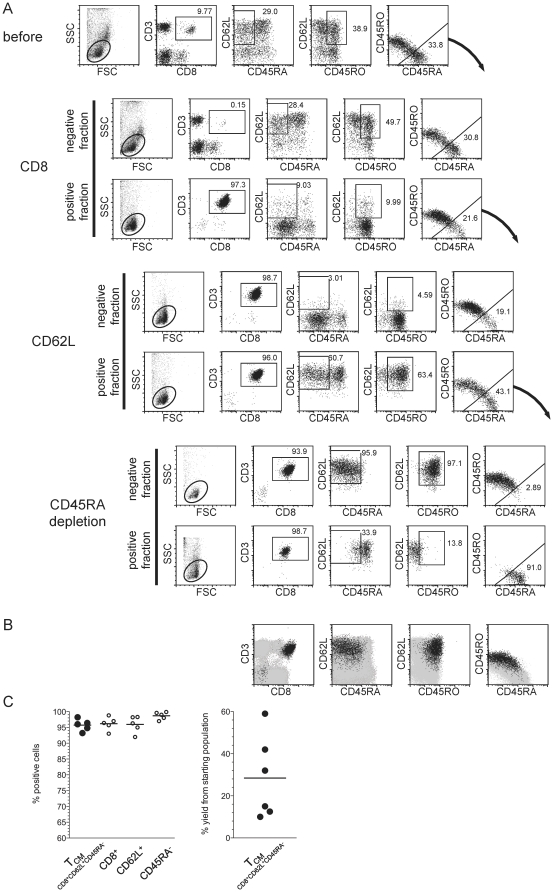
Serial magnetic cell enrichment of central memory T cells. (a) Serial magnetic enrichment of CD8^+^CD62L^+^CD45RA^neg^ central memory T cells from fresh PBMCs. Cells were first incubated with anti-CD8 Fab-multimers conjugated with *Strep*-Tactin-functionalized magnetic beads in order to pre-select CD8^+^ cells. The resulting positive fraction was then treated with D-biotin and washed to remove all anti-CD8 reagents. In a second step, CD62L positive T cells were enriched from the pre-selected CD8^+^ T cell pool via specific anti-CD62L Fab bound to *Strep*-Tactin coated magnetic beads and subsequently liberated from the selection reagents as described above. In a final step CD45RA^+^ cells were depleted from the pre-enriched CD8^+^CD62L^+^ cell population using CD45RA specific Fab-multimers conjugated to *Strep*-Tactin-coated beads. Living lymphocytes in the respective fractions of each selection step are shown. One representative experiment from five independent blood donors is shown. (b) Overlay of the enriched CD8^+^CD62L^+^CD45RA^neg^ cell population (black dots) derived from serial magnetic selection as shown in (a) and the corresponding starting population (underlying grey dots). (c) Summary of cell purities obtained within each purification step of multiparameter magnetic bead-based T_CM_ purifications as performed in (a) with PBMCs derived from 5 different blood donors (left graph, mean values are indicated). In the right graph, yields (in %) of the target T_CM_s are shown; mean value is indicated. For all samples analyzed by flow cytometry, at least 50.000 events have been acquired.

To evaluate the potential to purify T_CM_ using reversible staining reagents, we generated Fab-Streptamers for the surface markers CD8, CD62L and CD45RA that exhibited fully reversible staining (Fig. S3). The purification procedure consisted of 3 steps, starting with 2 positive enrichment cycles for CD8 and CD62L, followed by one depletion cycle to eliminate CD45RA^+^ cells. The performance of the purification for each single step (including all negative and positive fractions) is summarized in Fig. 3a. In the example, the final cell product reached a high purity of >95% CD8^+^ CD45RO^+^/CD45RA^−^ CD62L^+^ T_CM_ (Fig. 3a, b), and these high purities of T_CM_ were obtained in independent experiments with PBMC derived from five different donors. The cell yields were very similar to the ‘non-reversible’ protocol (Fig. S4), averaging 25% although with high variability in different donors (Fig. 3b). This demonstrates that reversible Fab-multimers can be effectively implemented into serial enrichment procedures to reliably enrich T_CM_ to very high purities using only 3 reagents that fulfill already the criteria for cell separation under GMP conditions.

### Sequential magnetic enrichment of regulatory T cells

**Figure 4:**
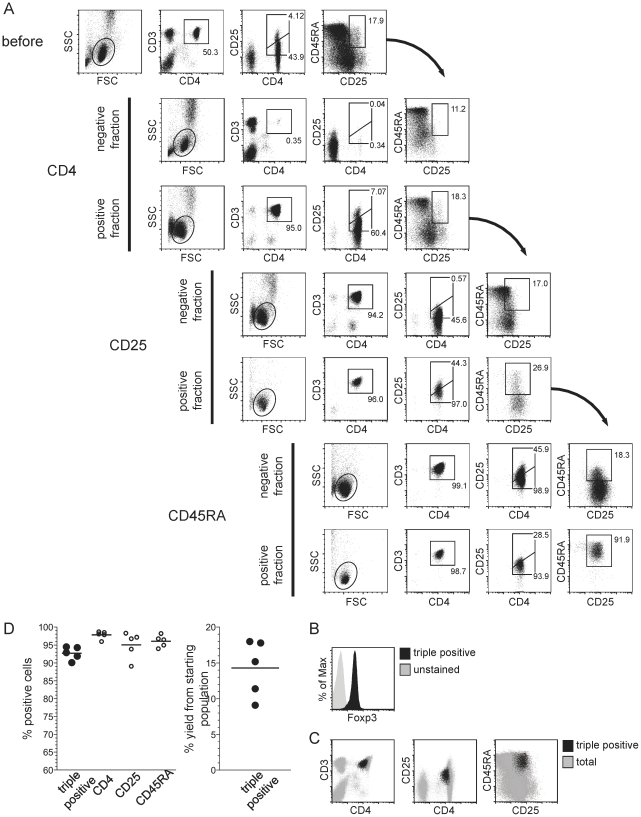
Serial magnetic cell enrichment of naturally occurring regulatory T cells. (a) Serial positive magnetic enrichment of CD4^+^CD25^+^CD45RA^+^ regulatory T cells (nTregs) from PBMCs. For pre-selection of CD4^+^ cells, PBMCs were first incubated with anti-CD4 Fab-multimers conjugated with *Strep*-Tactin-functionalized magnetic beads. The resulting positive fraction was then liberated from surface-bound label by D-biotin treatment and washed to remove anti-CD4 reagents. The second purification step comprised the selection for CD25 positive cells from the pre-selected CD4^+^ cell pool via specific anti-CD25 Fab bound to *Strep*-Tactin coated magnetic beads. Cell bound reagents were again removed from the resulting positive fraction by addition of D-biotin. In a third purification step, CD45RA^+^ cells were isolated from the enriched CD4^+^CD25^+^ cell population by using CD45RA-specific Fab-multimers conjugated to *Strep*-Tactin-coated magnetic beads. Living lymphocytes in the respective fractions of each selection step are shown. One representative experiment from five independent blood donors is shown. (b) Intracellular FoxP3 staining of triple positive enriched CD4^+^CD25^+^CD45RA^+^ regulatory T cells. (c) Overlay of the enriched CD4^+^CD25^+^CD45RA^+^ cell population (black dots) derived from serial magnetic selection as shown in (a) and the corresponding starting population (underlying grey dots). (d) Summary of cell purities obtained within each purification step of multiparameter magnetic bead-based nTregs purifications as performed in (a) with PBMCs derived from 5 different blood donors (left graph, mean values are indicated). In the right graph, yields (in %) of the target nTregs are shown; mean value is indicated. For all samples analyzed by flow cytometry, at least 50.000 events have been acquired.

Naturally occurring regulatory T cells (nTregs) have emerged as a promising population for therapy of autoimmune and alloimmune diseases. Therefore, isolation of minimally manipulated and highly functional Tregs represents a major goal for cell therapeutic approaches. Although there is debate on which marker combination is best for clinical nTreg purification, it is clear that only a combination of multiple markers can define this relatively rare cell subset. We generated extensive data demonstrating that the staining combination of CD4^+^, CD25^+^, CD45RA^+^ defines a highly pure nTreg population that maintains its phenotype and function even upon *in vitro* expansion to large cell numbers^14^. Currently, procedures for nTreg enrichment (for research use only) are based on the depletion of non-Treg cells using a complex antibody cocktail (including anti-CD8, CD14, CD16, CD19, CD36, CD56, CD123, TCRγδ, Glycophorin A, CD45RO, CD49d, CD127), followed by positive enrichment for CD25^+^ cells within the remaining cell population. Moreover, the large number of required antibodies makes transfer of this approach to clinical applications difficult. We speculated that serial positive enrichment could limit the reagents required for nTreg purification to just three (CD4, CD25, CD45RA). In addition to the already described reversible reagents for CD4 and CD45RA (Fig. 2 and 3), we completed the panel by generation of a reversible anti-CD25 Fab-fragment (Fig. S3b). Fig. 4a summarizes the first serial positive enrichment protocol with reversible reagents for these three different markers (including all negative and positive fractions). The panel of control stainings nicely demonstrates that after each purification step the positive selection marker must have been completely removed, as the following enrichment does not show any enrichment bias towards the previously used marker. In the example, a high purity of >90% CD4^+^ CD45RA^+^ CD25^+^ cells was achieved in the final product (Fig. 4a, d), and these cells homogeneously expressed Foxp3 (Fig. 4b). Such high purities of nTreg preparations were reproducibly obtained in independent experiments using PBMCs derived from five different donors. Yields often exceeded the expectation of 12.5% when considering a cell loss of approximately 50% per enrichment step (Fig. 4). Performing suppression assays as described previously [14], nTregs purified with reversible reagents were characterized by potent suppressive activity on *in vitro* stimulated responder T cells (Fig. S5). We believe that this first example of a triple serial positive enrichment protocol demonstrates the potential of the novel reversible Fab-mutlimer technology for isolation of low frequency cell subsets that can only be distinguished by multiple markers.

## Discussion

We describe here the development of a novel, fully reversible cell staining platform that enables serial positive enrichment over multiple cell surface markers. Our data shows the utility of this platform for the purification of CD8^+^ central memory T cells (T_CM_) and

naturally occurring regulatory T cells (nTregs), illustrating the potential of Fab-multimers for clinical cell separation in immunotherapy, and to overcome limitations of current techniques.

Most clinical cell sorting applications are currently based on reagents conjugated to paramagnetic beads. Very small beads, in the nano-particle range, are used for positive enrichment of desired cell populations for clinical application, the best example being CD34^+^ stem cells [28]. However, the relatively long duration of these processing procedures, the requirement for specialized equipment (reagents, columns, instruments), and potential problems caused by remaining bead conjugates on positively enriched cell populations are limitations. Larger beads (in the µm range), which can be used with technically less complicated and rapid cell processing procedures, are currently in clinical use only for cell depletion, because the co-transfer of larger beads into patients entails significant risk. The reversible multimer technology overcomes this problem, since the bead conjugates can be readily and fully removed from the purified cell population, independent of their size. In fact, all purification procedures described in this report were performed with paramagnetic beads of 1 µm in diameter, and marker/bead binding cells were retained using a permanent magnet in close proximity to the cell solution. This procedure can easily be transferred to different cell processing devices, including collection bags that are often used for clinical cell therapy. Therefore, we believe that the “*Strep*tamers” not only provides a novel option for purifying defined cell populations by serial positive enrichment, but also has the potential to vastly simplify and accelerate clinical cell processing procedures.

Multiparameter cell selection is a domain of flow cytometry-based cell sorting, but there is still a lack of routine applicability for clinical use. In a few locations worldwide, scientific groups and companies in close exchange with local authorities are currently installing flow cytometry cell sorters into GMP facilities (e.g. the Influx from BD) paving the way for upcoming first clinical trials. However, there are still many technical obstacles for bringing flow cytometry into clinical cell sorting, with one of the most prominent being remaining surface bound fluorochrome-conjugated staining reagents left on the purified cell population. A major advantage of reversible staining for clinical applications is that it avoids the co-infusion of labeling reagents such as monoclonal antibodies, fluorochromes or magnetic beads into patients. The recent fatalities that occurred following administration of a stimulatory CD28 superagonist illustrate the potential for unanticipated adverse events, and have made it essential that reagents with the potential to bind cell surface molecules that can alter cell function undergo laborious and costly pre-clinical *in vivo* testing for regulatory approval [29]. Reversible staining with Fab-multimers can also overcome side effects that might be mediated by the labeling regents themselves, even when signaling molecules are the targets for cell selection ([30], [31] and data not shown). The prevention of cell changes as a consequence of binding cell surface markers, which are even more difficult to monitor and predict when various labels are combined and bound to cells, is of general importance not only for clinical cell sorting, but also for interpreting the results of basic research studies that involve the transfer of functional cell populations. The (one dimensional) reversible MHC-*Strep*tamer [3] purification of antigen-specific T cells for clinical adoptive therapy was recently approved by European (European Medicines Agency) and German (Paul-Ehrlich-Institute) regulatory authorities as ‘minimally manipulated’, because when used under quality-controlled cell processing protocols, complete removal of the labeling reagents from the cell product was reliably demonstrated. Since the novel Fab-multimers described in the present study fulfill identical criteria, it is very likely that cell populations purified by serial positive enrichment will similarly be classified as ‘minimally manipulated’. This would substantially facilitate the implementation of multiparameter cell selection by serial positive enrichment into clinical applications.

## Supporting Information

Figure S1 **Organization of the Fab encoding operon.** Schematic overview of the operon encoding antibody Fab-fragments. The first cistron encodes the chimeric heavy chain, consisting of the respective V_H_ fragment, the human IgG1 constant domain and the C-terminal OneSTrEPtag. The V_H_ domain is N-terminally fused to the ompA signal peptide. The second cistron encodes the V_L_ domain N-terminally fused to the phoA leader peptide and C-terminally followed by the human κ constant domain.
(TIFF)

Figure S2 **Reversibility of Fab-multimer staining.** Western blot analysis of the removal of Fab-multimers generated with different anti-CD4 Fab-mutants (a) or the anti-CD25 Fab mutant (b). PBMCs were incubated with the respective CD4 or CD25 Fab-multimers, and following D-biotin treatment and subsequent washing, the cells were lysed, and parts of the liquid and pellet fractions were analyzed for remaining Fab-monomers using highly specific anti-OneSTrEPtag antibodies. The direct application of purified Fab-protein (a, first lane) served as a loading control. Flow cytometry-based control multimer stainings (solid line) compared to unstained cells (tinted histogram) are shown below to demonstrate that cells had been properly stained before dissociation.
(TIFF)

Figure S3 **Reversible staining by CD8, CD25, CD45RA and CD62L Fab-multimers.** FACS analysis of freshly isolated PBMCs stained with PE-labeled anti- CD8 (a), anti-CD25 (b), anti-CD45RA (c) and anti-CD62L Fab-multimers. Cells were analyzed either before (first column) or after (second column) treatment with D-biotin. After subsequent washing steps, remaining Fab-monomers were detected using (uncomplexed) PE-labeled *Strep*-Tactin (third column). Secondary Fab-multimer staining of reversibly stained cells served as control for successful removal of D-biotin (right column). Live CD3^+^ T cells (a, b and d) or CD14^−^CD19^−^ cells (c) are shown. The numbers in dot plots indicate the percentage of cells within gates.
(TIFF)

Figure S4 **Enrichment of T_CM_ using sequential depletion and positive selection of cells with non-reversible reagents.** a). PBMC were labelled with clinical grade anti CD4, CD45RA and anti CD14 mAb conjugated to paramagnetic beads (Miltenyi Biotec), and the labelled cells were removed using the AutoMACS or CliniMACS device. CD62L^+^ cells were then enriched from the remaining depleted fraction by a subsequent positive selection with a clinical grade biotin conjugated anti-CD62L mAb (DREG56 clone, kindly provided by City of Hope Cancer Research Center) and anti-biotin microbeads (Miltenyi Biotec, Germany). The panels show staining of live cells for CD3, CD8, CD62L, CD45RA, CD45RO, CD4, and CD14 in PBMC (before), and in the depleted and positively selected fractions. In the example, the depleting antibodies were highly effective in removing CD4^+^, CD14^+^, and CD45RA+ cells, and CD8^+^ T_CM_ were enriched to 35% in the final cell product. The large fraction (55%) of CD3^−^CD8^−^ cells in the final cell product (inset) are CD13^+^CD62L^+^ cells that are not removed by the depletion cocktail and are consistent with basophils based on staining with an extended panel of antibodies. b) Overlay of the enriched CD8^+^CD62L^+^CD45RA^neg^ cell population (black dots) after the two-step selection and the corresponding starting population (underlying grey dots). c) Summary of purity and yield of CD8^+^ T_CM_ from multiple experiments. The frequency of CD8^+^ T_CM_ in PBMC (bold) and after enrichment (open) for each donor is indicated by a circle. The phenotype of the major contaminating cells (CD8^+^CD62L^−^ and CD13^+^CD62L^+^) in the cell product is shown.
(TIFF)

Figure S5 **Functionality of Fab-multimer-isolated regulatory T cells.** Suppressive activity of regulatory T cells enriched by serial Fab-multimer staining (see Fig. 4). Suppression was determined after polyclonal stimulation in a CFSE dilution assay at a Treg∶T responder cell ratio of 2∶1, *n* = 3. Sort purities were usually above 95%. The vertical line separates divided from undivided cells.
(TIFF)
