# Supplementary figures and images for: Novel Serial Positive Enrichment Technology Enables Clinical Multiparameter Cell Sorting
Source: PLoS One. 2012 Apr 24;7(4):e35798. doi: 10.1371/journal.pone.0035798 (PMC3335788; doi:10.1371/journal.pone.0035798)

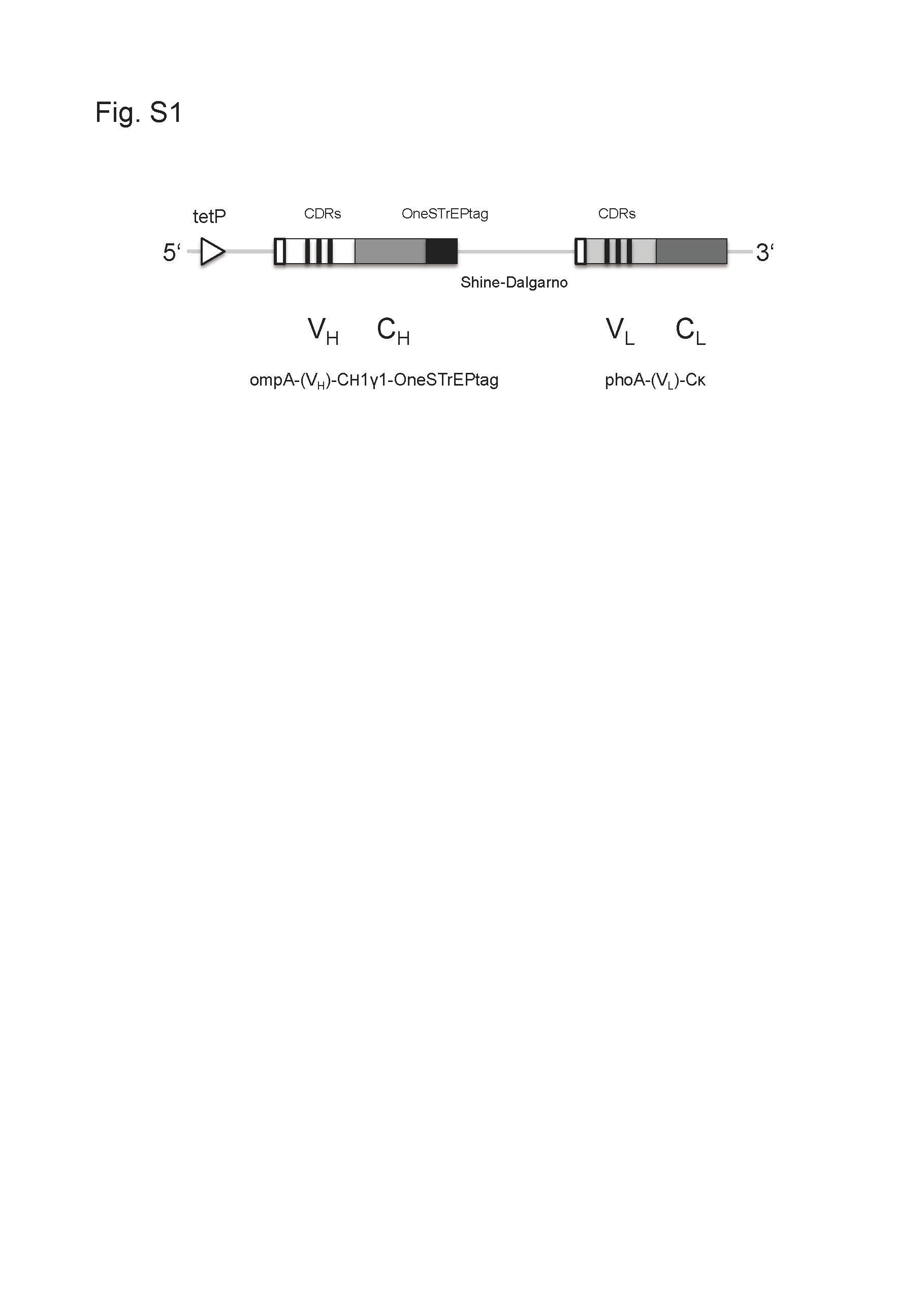

Supplement: Figure S1 — Organization of the Fab encoding operon. Schematic overview of the operon encoding antibody Fab-fragments. The first cistron encodes the chimeric heavy chain, consisting of the respective VH fragment, the human IgG1 constant domain and the C-terminal OneSTrEPtag. The VH domain is N-terminally fused to the ompA signal peptide. The second cistron encodes the VL domain N-terminally fused to the phoA leader peptide and C-terminally followed by the human κ constant domain. (TIFF) [file pone.0035798.s001.tiff]

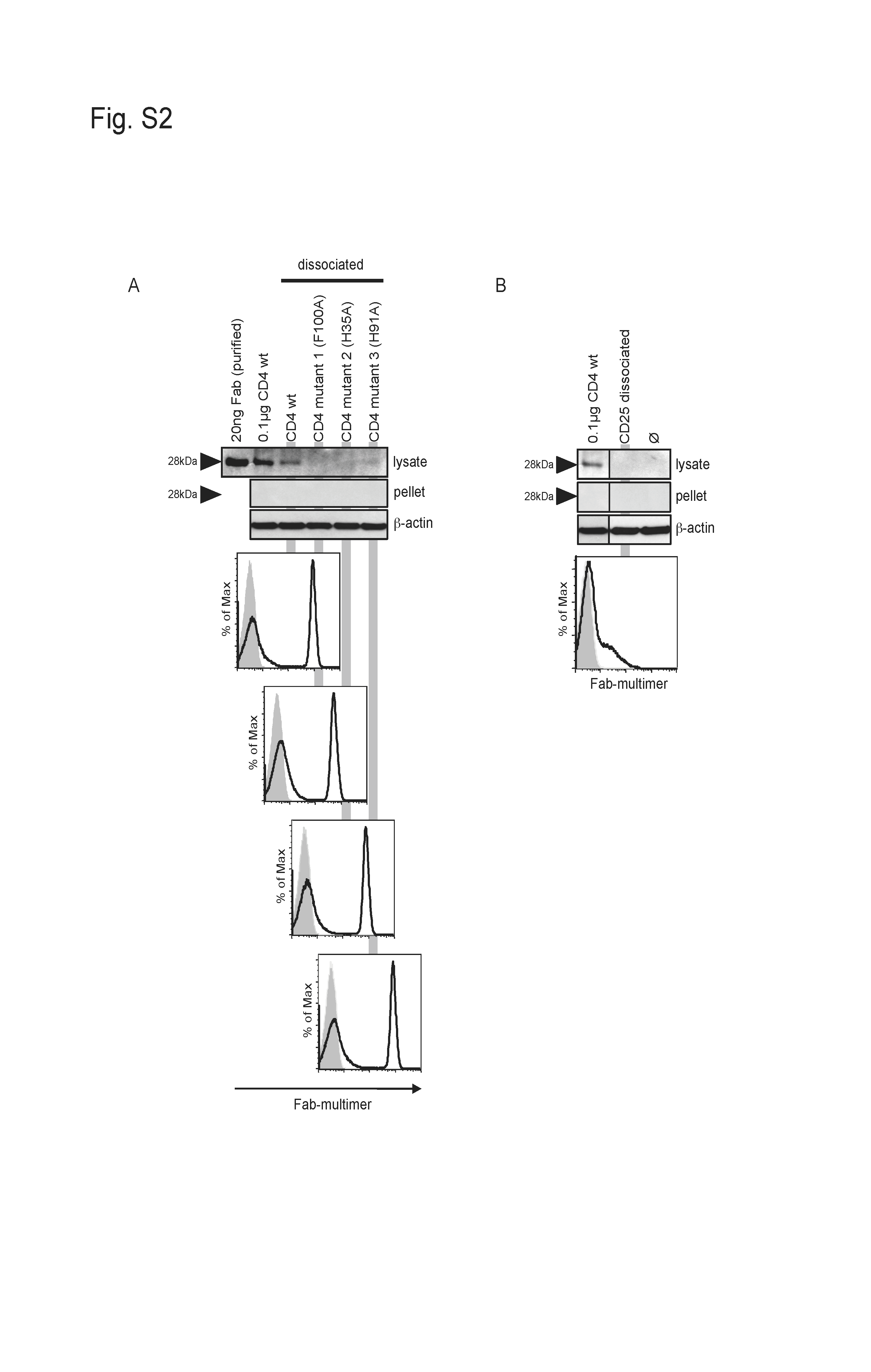

Supplement: Figure S2 — Reversibility of Fab-multimer staining. Western blot analysis of the removal of Fab-multimers generated with different anti-CD4 Fab-mutants (a) or the anti-CD25 Fab mutant (b). PBMCs were incubated with the respective CD4 or CD25 Fab-multimers, and following D-biotin treatment and subsequent washing, the cells were lysed, and parts of the liquid and pellet fractions were analyzed for remaining Fab-monomers using highly specific anti-OneSTrEPtag antibodies. The direct application of purified Fab-protein (a, first lane) served as a loading control. Flow cytometry-based control multimer stainings (solid line) compared to unstained cells (tinted histogram) are shown below to demonstrate that cells had been properly stained before dissociation. (TIFF) [file pone.0035798.s002.tiff]

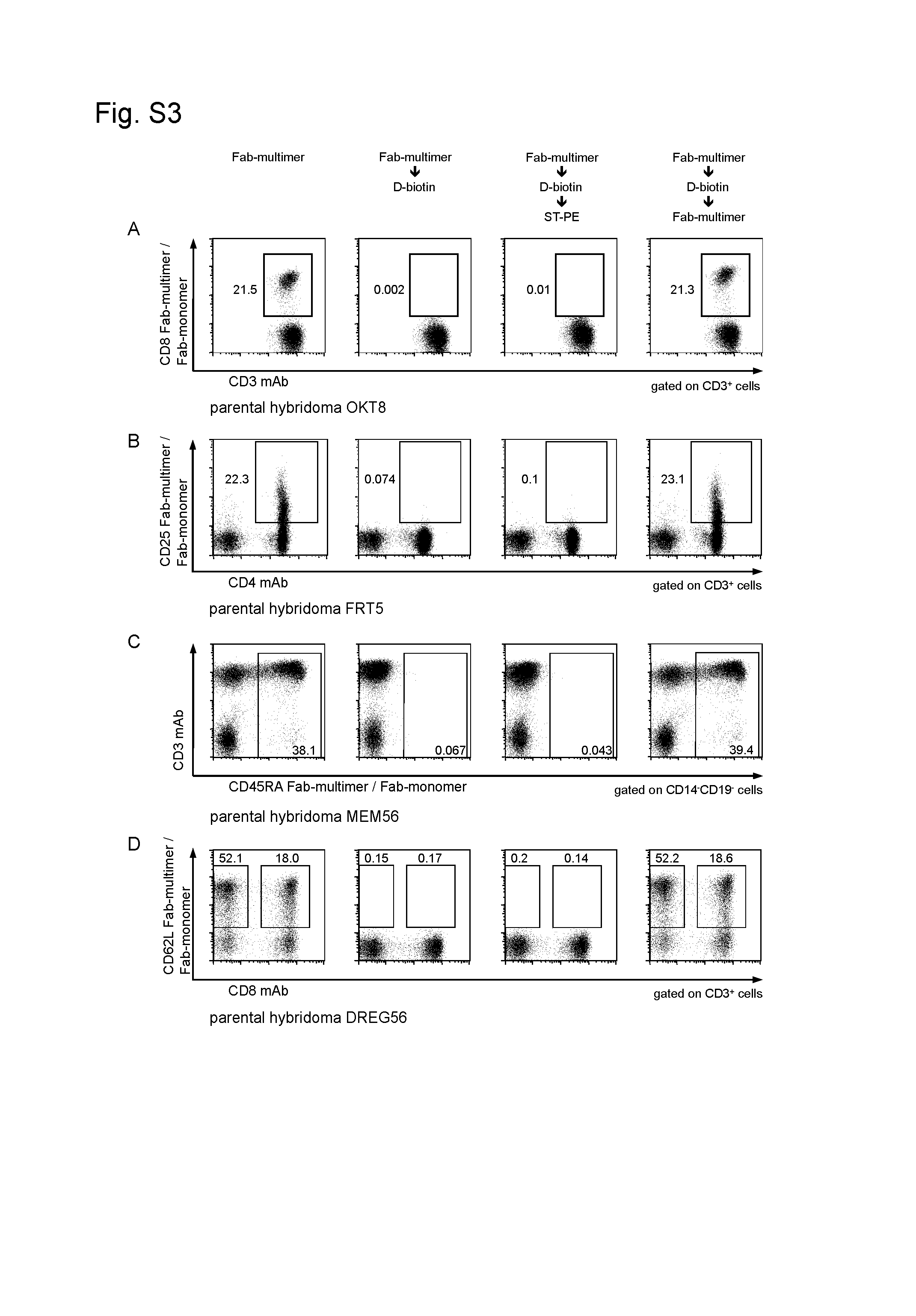

Supplement: Figure S3 — Reversible staining by CD8, CD25, CD45RA and CD62L Fab-multimers. FACS analysis of freshly isolated PBMCs stained with PE-labeled anti- CD8 (a), anti-CD25 (b), anti-CD45RA (c) and anti-CD62L Fab-multimers. Cells were analyzed either before (first column) or after (second column) treatment with D-biotin. After subsequent washing steps, remaining Fab-monomers were detected using (uncomplexed) PE-labeled Strep-Tactin (third column). Secondary Fab-multimer staining of reversibly stained cells served as control for successful removal of D-biotin (right column). Live CD3+ T cells (a, b and d) or CD14−CD19− cells (c) are shown. The numbers in dot plots indicate the percentage of cells within gates. (TIFF) [file pone.0035798.s003.tiff]

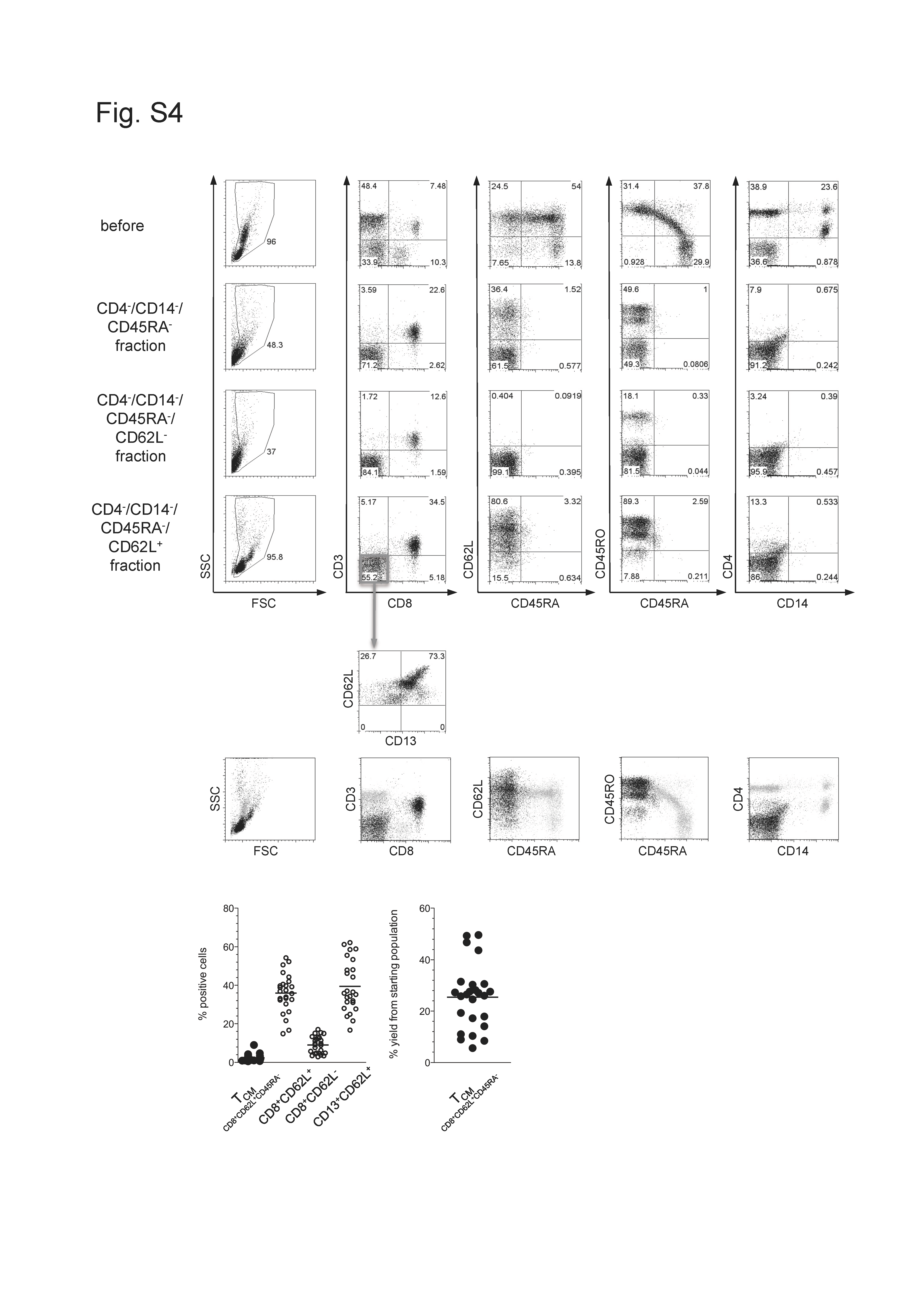

Supplement: Figure S4 — Enrichment of TCM using sequential depletion and positive selection of cells with non-reversible reagents. a). PBMC were labelled with clinical grade anti CD4, CD45RA and anti CD14 mAb conjugated to paramagnetic beads (Miltenyi Biotec), and the labelled cells were removed using the AutoMACS or CliniMACS device. CD62L+ cells were then enriched from the remaining depleted fraction by a subsequent positive selection with a clinical grade biotin conjugated anti-CD62L mAb (DREG56 clone, kindly provided by City of Hope Cancer Research Center) and anti-biotin microbeads (Miltenyi Biotec, Germany). The panels show staining of live cells for CD3, CD8, CD62L, CD45RA, CD45RO, CD4, and CD14 in PBMC (before), and in the depleted and positively selected fractions. In the example, the depleting antibodies were highly effective in removing CD4+, CD14+, and CD45RA+ cells, and CD8+ TCM were enriched to 35% in the final cell product. The large fraction (55%) of CD3−CD8− cells in the final cell product (inset) are CD13+CD62L+ cells that are not removed by the depletion cocktail and are consistent with basophils based on staining with an extended panel of antibodies. b) Overlay of the enriched CD8+CD62L+CD45RAneg cell population (black dots) after the two-step selection and the corresponding starting population (underlying grey dots). c) Summary of purity and yield of CD8+ TCM from multiple experiments. The frequency of CD8+ TCM in PBMC (bold) and after enrichment (open) for each donor is indicated by a circle. The phenotype of the major contaminating cells (CD8+CD62L− and CD13+CD62L+) in the cell product is shown. (TIFF) [file pone.0035798.s004.tiff]
